# Efficacy of Fetal MSC Therapy for Osteogenesis Imperfecta: A Systematic Review and Meta‐Analysis in Mice

**DOI:** 10.1155/sci/7815746

**Published:** 2026-09-25

**Authors:** Rakila Kurugalage, Aditya Mukherjee, Arian Bhatia, Ruurd L. Jaarsma, Krasimir Vasilev, Richard Bright, Vi-Khanh Truong

**Affiliations:** ^1^ Biomedical Nanoengineering Laboratory, Flinders University, Adelaide, Australia, flinders.edu.au; ^2^ Department of Orthopaedic and Trauma Surgery, Flinders Medical Centre, Flinders University, Adelaide, Australia, flinders.edu.au

**Keywords:** bone fragility, fetal mesenchymal stem cells, meta-analysis, osteogenesis imperfecta, regenerative therapy, stem cell transplantation

## Abstract

**Background:**

Osteogenesis imperfecta (OI) is a genetic disorder marked by collagen defects that cause skeletal fragility. Current treatments improve bone density but not bone quality or fracture rates. Fetal mesenchymal stem cells (fMSCs) show superior osteogenic and immunomodulatory potential compared with adult mesenchymal stem cells (MSCs) and may offer a disease‐modifying therapy.

**Methods:**

Following the Preferred Reporting Items for Systematic Reviews and Meta‐Analyses (PRISMA) 2020 guidelines, we systematically reviewed preclinical murine studies assessing the use of fMSC transplantation for OI. Data from eligible studies were pooled using random‐effects meta‐analysis (Hartung–Knapp adjustment). Risk ratios (RRs) were used for fracture incidence and standardized mean differences (SMDs) for mechanical and structural outcomes.

**Results:**

Four studies (219 mice: 91 treated, 128 controls) met inclusion criteria. fMSC treatment significantly reduced total long‐bone fracture incidence (RR = 0.30; 95% confidence interval [CI]: 0.13–0.72; *p* = 0.027). Nonsignificant trends favored fMSC therapy for maximum load (SMD = 0.60), bone volume fraction (SMD = 0.40), cortical thickness (SMD = 0.65), and stiffness (SMD = 0.57). Heterogeneity among secondary outcomes reflected methodological variability and differences in engraftment efficiency.

**Conclusions:**

fMSC transplantation significantly reduces fracture burden and shows consistent trends toward improved bone structure and strength in OI murine models. These findings support the need for further standardized, multicenter studies to optimize cell delivery, enhance engraftment, and validate clinical efficacy in humans. However, given the small evidence base, lack of independent replication, and reliance on a single OI mouse model, these findings should be interpreted as exploratory and hypothesis‐generating rather than confirmatory.

## 1. Introduction

Osteogenesis imperfecta (OI) is a genetic disorder characterized by altered bone tissue material properties, abnormal micro‐ and macro‐architecture, and increased bone turnover, resulting in bone fragility, frequent fractures, pain, and skeletal deformities [1]. The severity of OI varies widely, ranging from perinatal lethality to progressively deforming bone disease that may leave individuals permanently wheelchair‐bound with significant life‐limiting comorbidities. OI is a multisystemic condition, affecting not only the skeleton but also soft tissues, including tendons, ligaments, and the heart’s valvular rings, as well as the teeth and hearing [2]. The estimated incidence of OI is reported to affect 1 in 10,000 to 20,000 live births worldwide, with an estimated prevalence of 25,000 to 50,000 cases in the United States alone [3, 4]. The prevalence of OI in Australia is estimated to be about 1 in 20,000 to 1 in 30,000 live births [5]. Although its overall frequency is relatively rare, the considerable number of cases underscores the need for ongoing research and accessible treatment options.

OI is primarily caused by mutations in the COL1A1 and COL1A2 genes, which encode type I collagen, a crucial component of bone structure [6]. The disorder is further compounded by key bone remodeling pathway disruptions, including WNT, RANKL/RANK, TGF‐β, and MAPK signaling, which regulate the balance between bone formation and resorption [7]. Current therapeutic options, including bisphosphonates, anabolic agents, and antiresorptive therapies like denosumab, focus on improving bone density and reducing fracture rates but fail to address the underlying genetic defects or fully restore bone strength and quality [8]. These limitations underscore the need for innovative therapies that can target the root causes of the disease. Fetal mesenchymal stem cells (fMSCs) represent a promising disease‐modifying regenerative therapy for OI, aiming to improve bone formation and function rather than simply managing symptoms. Unlike gene‐editing or gene‐replacement approaches, fMSC therapy does not correct the underlying COL1A1 or COL1A2 mutations responsible for abnormal collagen synthesis. Instead, transplanted cells contribute healthy extracellular matrix, enhance osteogenesis, and modulate the bone microenvironment through paracrine and immunomodulatory mechanisms, thereby reducing fracture risk and improving bone quality.

Unlike adult‐derived mesenchymal stem cells (MSCs), fMSCs exhibit superior regenerative potential, enhanced osteogenic differentiation capacity, and a more robust ability to modulate bone remodeling processes [9–11]. They also possess immunomodulatory properties, making them promising candidates for transplantation with minimal risk of immune rejection [12, 13]. The therapeutic rationale for fMSC transplantation in OI lies in its dual capacity to restore osteogenic potential and modulate the pathological bone microenvironment. Beyond replacing defective osteoblasts, fMSCs exert both direct and indirect regenerative effects. They can differentiate into osteoblast‐like cells; secrete osteogenic cytokines and growth factors such as BMP2, VEGF, and IGF‐1; and release extracellular vesicles enriched with pro‐osteogenic microRNAs that enhance bone formation and suppress osteoclastic activity. Moreover, their immunomodulatory secretome attenuates the production of inflammatory cytokines (e.g., TNF‐α and IL‐6), thereby reducing aberrant bone remodeling and promoting balanced turnover. These combined mechanisms suggest that fMSC therapy may produce paracrine‐driven improvements in bone quality even when engraftment efficiency remains low. Therefore, by integrating preclinical evidence from multiple experimental paradigms, this review aims to quantitatively and qualitatively evaluate whether fMSC transplantation can meaningfully reduce fracture risk and enhance bone integrity in murine models of OI. We assess the impact of fMSCs on bone‐related outcomes, specifically fracture incidence, mechanical strength factors (maximum load and stiffness), and structural outcomes, including cancellous bone volume fraction (BV/TV) and cortical thickness. The findings aim to inform the translational optimization of stem cell–based therapies, including cell sourcing, delivery timing, and dosage strategies, to advance the development of safe and effective clinical applications.

## 2. Methods

### 2.1. Study Selection

**Figure 1:**
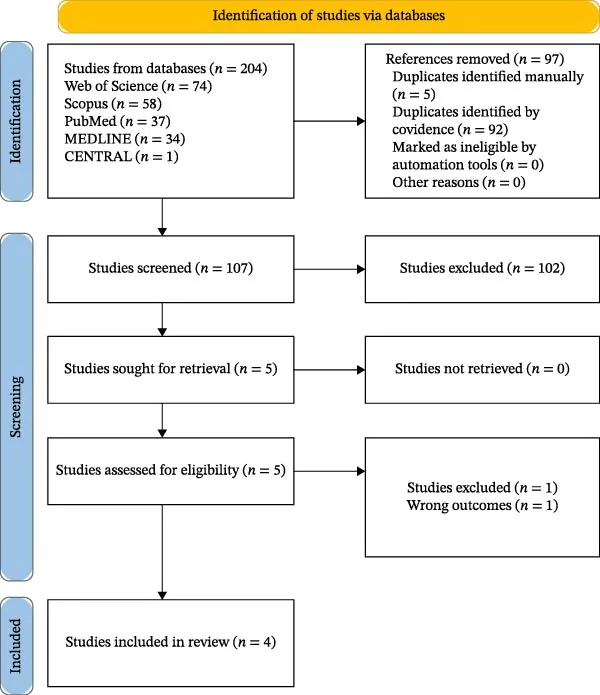
Preferred Reporting Items for Systematic Reviews and Meta‐Analyses (PRISMA) flow diagram.

This systematic review and meta‐analysis followed a prospectively developed protocol and is reported in accordance with the Preferred Reporting Items for Systematic Reviews and Meta‐Analyses (PRISMA) 2020 guidelines (Table S1) [14]. The search strategy included the terms (“Osteogenesis Imperfecta” OR “OI” OR “brittle bone disease” OR “brittle bones”) AND (“Fetal” OR “Foetal”) AND (“Mesenchymal Stem Cell” OR “MSCs” OR “Stromal Cell ^∗^”) were used to find articles published between 2000 and September 2025 on electronic databases, namely, PubMed/MEDLINE, Web of Science, Scopus, and CENTRAL. Search results were imported into the “Covidence” platform, and two reviewers independently screened titles, abstracts, and full texts using predefined eligibility criteria. Conflicts were resolved through discussion with the review team until consensus was achieved. The PRISMA flow diagram, summarizing this process, is provided in Figure 1.

### 2.2. Eligibility Criteria

Regarding study design, only controlled experimental studies on murine models were included. Clinical trials, case reports, and in vitro studies were excluded. Studies using animal models other than mice were also excluded to mitigate the risk of excessive heterogeneity. Studies using mouse populations with OI were eligible for inclusion, regardless of the specific strain or phenotypic severity of the disease. Studies providing fMSCs (derived from any source) as an intervention were included. Studies using fMSCs or non‐MSCs as interventions were excluded. It was also required that the control group consist solely of untreated mice.

### 2.3. Bone Outcome Analysis in fMSC Therapy for OI

This review focuses specifically on the efficacy of fMSCs on bone outcomes. Therefore, we collected data on key quantitative measures of bone quality commonly reported in preclinical studies. These were fracture incidence, which reflects the treatment’s impact on bone fragility; maximum load, quantifying the highest force a bone can endure before failure; stiffness, quantifying the bone’s resistance to deformation under stress; trabecular bone density as represented by the bone volume‐to‐total volume ratio (BV/TV); and cortical thickness. Only outcomes reported by two or more independent studies were included in the meta‐analysis, ensuring sufficient data for statistical pooling and reducing the effect of isolated findings on overall conclusions. When outcomes were recorded at different times, the most frequently reported measurement time across studies was used in the meta‐analysis.

### 2.4. Risk of Bias Assessment

Two independent reviewers assessed the risk of bias using SYRCLE’s tool for animal studies, and conflicts were resolved through discussion with the review team [15]. The assessment is provided in Figure S1. The domains appraised were selection (randomization, baseline balance, and allocation concealment), performance (random housing and caregiver/investigator blinding), detection (random outcome assessment and assessor blinding), attrition (missing data), reporting (selective reporting), and other biases.

### 2.5. Statistical Analysis

Meta‐analysis was performed in RStudio (version 4.5.1), using the “meta” package. All code and associated data are available on request. Given the limited number of included studies, a random‐effects inverse‐variance model with Hartung–Knapp adjustments and a restricted maximum–likelihood estimator for *τ*2 was used. Binary variables were pooled according to risk ratios (RRs) and continuous variables according to standardized mean difference (SMD). Heterogeneity among studies was assessed using the Cochran’s *Q* test and quantified using the *I* ^2^ statistic. Influence analysis used a leave‐one‐out (LOO) method with the same model as the original meta‐analysis. The result of any statistical analysis was considered significant if the *p*‐value was less than 0.05. Assessment of publication bias using funnel plots was not performed due to the limited number of included studies (<10).

## 3. Results

### 3.1. Included Studies

The initial search identified 204 records across five databases: Web of Science (74), Scopus (58), PubMed (37), MEDLINE (34), and CENTRAL (1). After removing 97 duplicates, 107 records were screened by title and abstract. Of these, 102 were excluded. Five full‐text articles were assessed for eligibility; none were unobtainable, and one was excluded for wrong outcomes. Ultimately, four studies [16–19] met the inclusion criteria and were included in the systematic review and meta‐analysis. A completed PRISMA checklist (Table S1) outlines the adherence of this study to each reporting criterion.

### 3.2. Characteristics of Included Studies

Across the four preclinical studies, a total of 219 mice with OI (genotype: B6C3Fe a/a—Col1a2^oim^/Col1a2^oim^) were included, with 91 of these receiving fMSC transplantation and 128 serving as untreated controls. Table 1 summarizes the characteristics of the four included studies. The human‐derived fMSCs utilized in these studies were sourced at 9–12 weeks from chorionic villous tissue, fetal blood, or amniotic fluid. The mice were treated by intraperitoneal (IP) injection of 1.0 × 10^6^ cells at either the neonatal or fetal stage of their development. Mice injected during the neonatal stage received treatment between postnatal day (PN) 3 and 4, and those injected during the fetal stage received treatment between embryonic day (E) 13.5 and 15.

**Table 1:** Characteristics of included studies.

Study	Year	Sample size, *E* (n)	Sample size, *C* (n)	Genotype of mouse model used	fMSC source	Gestational age at which cells were sourced (weeks)	fMSC dose (×10^6^ cells)	Treatment delivery route	Stage of life at time of injection	Injection timing
Jones et al. [16]	2014	22	34	B6C3Fe·a/a—Col1a2^oim^/Col1a2^oim^	hCVT	9–10	1.0	IP injection	Neonate	PN3 to PN4
Guillot et al. [17]	2008	29	48	B6C3Fe·a/a—Col1a2^oim^/Col1a2^oim^	hFB	10	1.0	IP injection	Fetal	E13.5 to E15
Ranzoni et al. [18]	2016	28	26	B6C3Fe·a/a—Col1a2^oim^/Col1a2^oim^	hAF	12	1.0	IP injection	Neonate	PN3 to PN4
Vanleene et al. [19]	2011	12	20	B6C3Fe·a/a—Col1a2^oim^/Col1a2^oim^	hFB	10	1.0	IP injection	Fetal	E13.5 to E15

*Note:* Comparative overview of published studies detailing experimental design parameters, including sample sizes for experimental (E) and control (C) groups, mouse genotypes, fMSC sources, gestational age at cell isolation, administered cell doses, delivery routes, and injection timing relative to developmental stage.

Abbreviations: C, control group; E, embryonic day; E, experimental group; fMSCs, fetal mesenchymal stem cells; hAF, human amniotic fluid; hCVT, human chorionic villus tissue; hFB, human fetal blood; IP, intraperitoneal; n, number; oim, osteogenesis imperfecta murine; PN, postnatal day.

### 3.3. Fracture Incidence

**Figure 2:**
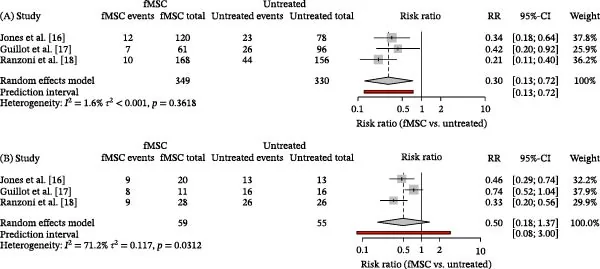
Meta‐analysis of fracture incidence outcomes. Forest plots display study‐level and pooled risk ratios (RRs) with 95% confidence and prediction intervals, comparing fracture incidence outcomes between OI mice transplanted with fMSCs and untreated OI mice. The fracture incidence outcomes included (A) total long‐bone fractures and (B) mice with ≥1 long‐bone fracture.

Across the studies by Jones et al. [16], Guillot et al. [17], and Ranzoni et al. [18] (*n* = 679 fractures), fMSC treatment reduced the total number of long‐bone fractures in comparison to no treatment, yielding a pooled RR = 0.30 (95% confidence interval (CI) 0.13–0.72; *p* = 0.027; *I* ^2^ = 1.6%) (Figure 2A). Results from the same trio, regarding the number of mice with ≥1 long‐bone fracture, were also more favorable in the fMSC group, with a pooled RR = 0.50 (95% CI 0.18–1.37; *p* = 0.098; *I* ^2^ = 71.2%) (Figure 2B), but failed to reach statistical significance. Vanleene et al. [19] did not report all long‐bone fractures; therefore, their study was excluded from these meta‐analyses. However, they found fewer femoral fractures in the treated OI mice group than in the untreated group (4.2% vs. 27.5%; *p* < 0.001).

### 3.4. Secondary Outcomes

#### 3.4.1. Maximum Load

**Figure 3:**
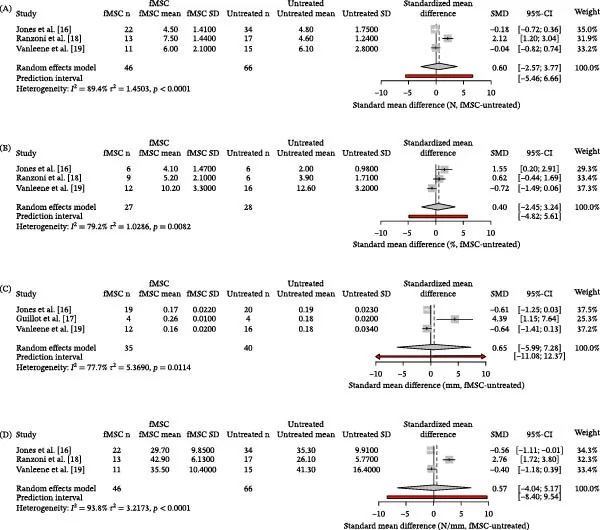
Meta‐analysis of secondary outcomes. Forest plots display study‐level and pooled standardized mean differences (SMDs) with 95% confidence intervals comparing secondary study outcomes in fMSCs transplanted into osteogenesis OI mice and untreated OI mice. Secondary outcomes included (A) maximum load, (B) trabecular bone volume fraction (BV/TV), (C) cortical thickness, and (D) stiffness.

Studies conducted by Jones et al. [16], Ranzoni et al. [18], and Vanleene et al. [19] (*n* = 112) showed that fMSC therapy showed a directionally positive but statistically nonsignificant effect on maximum load, yielding a pooled SMD of 0.60 (95% CI, −2.57 to 3.77; *p* = 0.501; *I* ^2^ = 89.4%), indicating a modest trend toward enhanced mechanical strength of the repaired bone in fMSC‐treated groups. However, the high heterogeneity across studies reflects substantial variation in experimental conditions, including the type of bone defect, the animal models used, delivery routes, and evaluation time points, which may have influenced the overall outcome. (Figure 3A).

#### 3.4.2. Bone Volume

Across three studies by Jones et al. [16], Ranzoni et al. [18], and Vanleene et al. [19] (*n* = 55), fMSC treatment showed a directionally positive but nonsignificant effect on BV/TV, yielding a pooled SMD = 0.40 (95% CI −2.45 to 3.24; *p* = 0.61; *I* ^2^ = 79.2%) (Figure 3B). In summary, while these preclinical results suggest a potential benefit of fMSC administration in enhancing bone formation, the lack of statistical significance and substantial interstudy heterogeneity highlight the need for more standardized experimental approaches and larger, well‐controlled studies to clarify the therapeutic potential of fMSCs in bone tissue repair.

#### 3.4.3. Cortical Thickness

Studies by Jones et al. [16], Guillot et al. [17], and Vanleene et al. [19] (*n* = 75) showed that fMSC treatment again showed a directionally positive but nonsignificant effect on cortical thickness, yielding a pooled SMD of 0.65 (95% CI −5.99 to 7.28; *p* = 0.72; *I* ^2^ = 77.7%), suggesting a modest trend toward cortical bone enhancement in fMSC‐treated groups. However, this effect did not reach statistical significance, and the substantial interstudy heterogeneity highlights the variability in experimental design, including differences in animal models, defect sites, dosage regimens, and time points of evaluation (Figure 3C).

#### 3.4.4. Stiffness

In studies conducted by Jones et al. [16], Ranzoni et al. [18], and Vanleene et al. [19] (*n* = 112), fMSC treatment showed a directionally positive but nonsignificant effect on stiffness, yielding a pooled SMD = 0.57 (95% CI −4.04 to 5.17; *p* = 0.649; *I* ^2^ = 93.8%). This suggests a mild tendency toward increased mechanical rigidity in the fMSC‐treated groups compared to the controls. However, the wide CI and substantial heterogeneity indicate considerable variability among studies, reflecting differences in animal models, fracture sites, cell doses, delivery routes, and assessment methods (Figure 3D). The notable variability in secondary biomechanical and structural outcomes likely results from methodological differences across studies, including variations in animal age, skeletal sites examined, assessment methods, and measurement timing. As a result, these pooled analyses were viewed more as indicators of directional consistency rather than sources of precise effect estimates.

### 3.5. Engraftment Variability Across Studies

**Figure 4:**
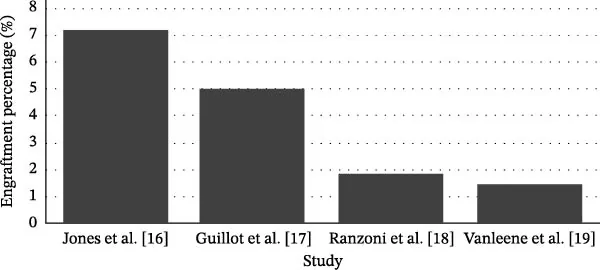
Engraftment of fMSCs in bone tissue across the included studies. Bars represent the highest reported donor‐cell engraftment within skeletal tissue (bone) in each study following transplantation. Engraftment was assessed using study‐specific histological and molecular detection methods; therefore, values should be interpreted as comparative rather than directly equivalent.

Among these studies, Jones et al. [16] reported the highest engraftment rate, ~7%, suggesting superior retention and integration of transplanted fMSCs within the host tissue. Guillot et al. [17] reported an engraftment of around 5%, whereas Ranzoni et al. [18] and Vanleene et al. [19] demonstrated considerably lower engraftment efficiencies, with values below 2% (Figure 4). The dissociation between low engraftment efficiency and consistent fracture reduction supports a predominantly paracrine and immunomodulatory mechanism rather than direct structural cell replacement.

## 4. Discussion

### 4.1. Study Findings

Despite using a random‐effects model to pool data from a few studies, the meta‐analysis demonstrated a statistically significant 70% risk reduction in long‐bone fracture incidence with fMSC treatment compared to no treatment. Fracture incidence likely represents a more sensitive and clinically meaningful endpoint in OI than isolated biomechanical or microstructural parameters, particularly given the heterogeneity of preclinical study designs.

There was also a 50% reduction in the proportion of mice with at least one long‐bone fracture; however, this metric did not reach statistical significance. Biomechanical and structural endpoints, such as maximum load, BV/TV, cortical thickness, and stiffness, trended in favor of fMSC treatment but did not reach statistical significance. LOO sensitivity analyses did not produce any statistically significant results. For secondary outcomes, omitting a single study frequently reversed the direction of the SMD and reduced heterogeneity to zero or near‐zero (Tables S2–S7). Reviewing individual study methods, no obvious source of these discrepant results could be identified. The wide prediction intervals suggest that underlying methodological differences may explain the between‐study heterogeneity and indicate uncertainty in the results obtained for these variables, rather than a lack of treatment efficacy. Accordingly, meta‐analyses of secondary biomechanical and structural outcomes were conducted to assess directional consistency rather than to generate definitive pooled effect estimates.

Beyond differences in sourcing, dosing, and treatment timing, outcome‐specific methods also varied. Subgroup analysis by treatment timing (prenatal versus neonatal) was not feasible due to the limited number of eligible studies; however, both approaches consistently favored a reduction in fracture burden, suggesting a shared disease‐modifying mechanism. For example, measurement modalities for factors such as cortical thickness and BV/TV included microcomputed tomography, X‐ray microradiography, and histomorphometry. Also, the reported rate of fMSCs engraftment ranged between 1.4% and 7.1% across studies. Despite variable engraftment rates, the consistent reduction in fracture risk suggests that clinical benefit may be mediated by durable cellular integration and paracrine mechanisms. Overall, these preclinical signals are promising and justify larger, well‐powered clinical trials in humans. The following section summarizes the limited but growing clinical literature.

### 4.2. Current Clinical Evidence for fMSC Transplantation in OI Patients

Limited but encouraging clinical evidence suggests that fetal MSC therapy can modify the course of OI, most convincingly when administered as prenatal (in utero) therapy, followed by postnatal booster infusions [13, 20, 21]. In the earliest human case, intrauterine infusion at 32 weeks led to donor‐cell chimerism detected at 9 months (median 7.4% by whole‐Y fluorescence in situ hybridization) median 7.4%, the formation of organized bony trabeculae, normal psychomotor development, and only three fractures within the first 2 years of life, despite severe prenatal disease [13]. In a two‐center experience, a child with type III OI received prenatal fMSCs, followed by a same‐donor booster at 8 years, and showed improvements in linear growth, mobility, and fracture incidence despite low‐level bone engraftment. A second fetus with type IV OI, infused intravenously at 31 weeks, had no new fractures for the remainder of the pregnancy or during infancy. Growth tracked normally until 13 months, slowed thereafter, and resumed following a booster infusion at 19 months [20].

The TERCELOI trial followed two children with OI for 2.5 years, with five fMSC infusions at 5‐to‐6‐month intervals [21]. Annual fractures declined from 8 to 3 in patient 1 and from 2 to 0 in patient 2. Lumbar spine bone mineral density (BMD) increased by +0.043 and +0.023 g/cm^2^ in the early and late phases, respectively, from a baseline of 0.336 g/cm^2^ in patient 1, with similar gains in patient 2. Trabecular microarchitecture improved with higher BV/TV and early reductions in trabecular separation, particularly after the first two infusions. No treatment‐related adverse events or activation of patient peripheral blood mononuclear cells (PBMCs) against donor MSCs were detected, and immunophenotyping remained stable [21].

Route and timing are important, with prenatal (in utero) transplantation generally used as the initial treatment, followed by postnatal IV booster infusions to reinforce therapeutic benefit [13, 20, 21]. Two clinical trials are currently underway to further establish these findings. The multicenter BOOSTB4 phase I/II trial administers four IV doses of first‐trimester fetal‐liver MSC product at 4‐month intervals and tracks fractures, growth, BMD, and turnover biomarkers for up to 10 years [22]. Furthermore, a complementary exploratory program, BOOST2B, evaluates repeated IV and intraosseous delivery of the same MSC product in children aged 1–4 years, with safety/tolerability as the primary endpoint and fracture count and time‐to‐first‐fracture as key secondary endpoints [23]. While direct clinical extrapolation from murine models is not possible, the concordance between preclinical fracture reduction and early human clinical signals strengthens the biological plausibility of fetal MSC therapy as a disease‐modifying intervention.

Overall, current clinical reports demonstrate a reduced fracture burden, consistent with the meta‐analysis’s findings, as well as measurable gains in BMD and microstructural improvements, without clinically significant immune rejection [21]. Before routine clinical use, safety and efficacy must be confirmed through prospective human clinical trials, and treatment timing, dosing, and delivery must be optimized. The BOOSTB4 and BOOST2B trials are designed to further these goals [22, 23]. While these early clinical observations align with the preclinical fracture‐reduction signal identified in this meta‐analysis, direct extrapolation from murine models to human disease remains inappropriate without adequately powered prospective trials.

The present meta‐analysis demonstrated a significant 70% reduction in fracture incidence following fMSC therapy in murine OI models. Although direct quantitative comparison with human studies is not possible due to limited patient numbers and heterogeneous study designs, the available clinical evidence indicates a similar directional effect. Case reports, the TERCELOI study, and ongoing BOOSTB4 and BOOST2B trials consistently report reductions in fracture frequency, improvements in BMD and functional outcomes, and favorable safety profiles. Unlike the murine studies, human studies have not yet provided sufficient data for pooled quantitative analysis and have primarily focused on safety and feasibility. Furthermore, although engraftment in both mice and humans appears low, therapeutic benefits are observed in both settings, supporting the hypothesis that fMSCs act predominantly via paracrine and immunomodulatory mechanisms rather than direct structural replacement. Overall, the concordance between preclinical and early clinical findings strengthens the translational rationale for fetal MSC therapy while highlighting the need for larger, controlled clinical trials before definitive conclusions regarding efficacy can be drawn.

### 4.3. Limitations

This study has notable limitations to consider when interpreting the results. Although a meta‐analysis was possible, the current quantitative synthesis is more exploratory than confirmatory. Only four suitable preclinical studies were found, reflecting the limited evidence for fMSC therapy for OI rather than strict eligibility constraints. As a result, the combined effect estimates, especially for secondary biomechanical and structural outcomes, should be viewed with caution. Conservative random‐effects modeling with a Hartung–Knapp adjustment was used to address small‐study effects, but the small number of studies limited statistical power, resulting in wide CIs and uncertainty in several pooled estimates.

Second, the current evidence base lacks independent replication. All included studies were conducted by the same research group or closely collaborating investigators, reflecting the early stage of this research field. Although these studies spanned several years and used different fetal MSC sources and experimental protocols, the absence of independent validation introduces the possibility of investigator or network bias and limits confidence in the reproducibility and generalizability of the observed therapeutic effects. Independent multicenter preclinical studies are, therefore, required to confirm these findings.

Third, all included studies utilized the same murine model of OI (B6C3Fe a/a‐Col1a2^oim^/Col1a2^oim^), representing a single genetic subtype of the disease. Although this reduced biological heterogeneity within the meta‐analysis, it limits the applicability of the findings to the broader spectrum of OI, which comprises numerous genotypes and phenotypic severities. Future studies should evaluate fMSC therapy in additional clinically relevant OI models to determine whether the observed therapeutic effects are consistent across different genetic forms of the disease.

Furthermore, considerable methodological heterogeneity existed across studies, including differences in fetal MSC source (chorionic villus tissue, fetal blood, or amniotic fluid), timing of administration (prenatal versus neonatal), outcome assessment methods, follow‐up periods, and approaches for quantifying donor‐cell engraftment. These methodological differences likely contributed to the heterogeneity observed in several secondary outcomes and prevented meaningful subgroup analyses to determine the influence of individual treatment variables.

This review intentionally focused exclusively on fMSCs, as these cells possess distinct biological characteristics, including enhanced proliferative capacity, greater osteogenic potential, reduced senescence, and lower immunogenicity compared with perinatal or adult MSCs. The objective was, therefore, to evaluate the evidence supporting this specific therapeutic approach rather than to compare different MSC sources. Nevertheless, the absence of direct comparative studies means that it remains uncertain whether the observed therapeutic benefits are unique to fMSCs or represent a broader property of MSC therapy. Comparative studies evaluating fetal, perinatal, and adult MSCs are needed to address this important knowledge gap.

Finally, several studies incompletely reported methodological details, including randomization, allocation concealment, and blinding procedures, introducing potential risks of selection and performance bias. The small number of studies also precluded formal assessment of publication bias using funnel plots or meta‐regression analyses. Moreover, although murine models provide valuable mechanistic insights, they cannot fully replicate the clinical heterogeneity and complexity of human OI. The currently available clinical evidence consists primarily of case reports, small case series, and early‐phase clinical trials, which limit quantitative comparison with preclinical findings. Therefore, while the concordance between the animal studies and early clinical observations is encouraging, the present findings should be interpreted as preliminary and hypothesis‐generating until confirmed by larger, independently conducted preclinical studies and adequately powered prospective clinical trials.

### 4.4. Future Prospects for fMSC Therapy in OI

FMSC therapy for OI is promising, but its translation to clinical practice now hinges on larger, multicenter trials with diverse genotypes/phenotypes and longer follow‐up periods to validate early signals [13, 20, 21]. Future studies should utilize standardized eligibility criteria, treatment parameters, and outcome measurement methods to improve the reliability of subsequent evidence. Furthermore, trials should embed biomarker programs with serial sampling to identify paracrine mediators and osteogenic pathways [23]. Also, recent evidence suggests that stem cell delivery and engraftment may be improved by strategies such as genetic modification to upregulate CXCR4 expression [24]. Such strategies should be investigated, given the low and variable engraftment observed in preclinical evidence [16–19]. Adjunctive treatments and fMSC therapy to tackle OI’s inflammatory and mechanically dysregulated bone formation (e.g., osteoinductive growth factors and targeted anti‐inflammatory regimens) also warrant evaluation [25]. Many variations in treatment sources, doses, timing, frequency, and route have been studied and require optimization. This may be achieved through comparative studies investigating the effects of altering specific treatment parameters on overall efficacy. For example, studies may compare prenatal versus postnatal treatment administrations, given that early signals favor earlier interventions [13, 20]. The studies should also incorporate routine immune monitoring to detect sensitization and guide immunomodulatory support for allogeneic responses. These findings suggest that fracture incidence may represent a more sensitive and clinically relevant endpoint for OI.

## 5. Conclusion

This exploratory systematic review and meta‐analysis provide preliminary evidence that fMSC therapy may reduce fracture incidence and improve bone quality in murine models of OI. However, these findings should be interpreted with caution given the limited evidence base, the lack of independent replication, and the reliance on a single animal model. Larger, independently conducted preclinical studies and well‐designed clinical trials are required to confirm these observations. Across preclinical murine models, fMSC transplantation significantly reduced the incidence of long‐bone fractures and consistently improved bone structural and mechanical properties, even when statistical significance was not achieved. However, translation to clinical practice remains limited by low engraftment efficiency, methodological heterogeneity, and the absence of long‐term, standardized outcome assessment. These findings underscore the need for carefully designed, multicenter studies incorporating standardized protocols, harmonized reporting, and sufficient statistical power to validate preclinical results. Future investigations should focus on enhancing the homing, survival, and osteogenic potency of fMSCs through strategies such as genetic modification (e.g., upregulation of CXCR4), biomaterial‐assisted delivery, or coadministration with osteoinductive factors. Integrating immune monitoring and mechanistic biomarker analyses will further clarify the paracrine and immunomodulatory mechanisms underlying therapeutic efficacy. Ultimately, through coordinated efforts across stem cell biology, orthopedic research, and clinical translation, fMSC therapy may progress from experimental intervention to a viable, scalable treatment paradigm for patients with OI. This approach has the potential to move beyond symptomatic management by providing a regenerative, disease‐modifying strategy that complements existing therapies, although it does not correct the underlying genetic defect.

## Funding

The authors acknowledge the support of the Capstone Research Scholarship. Open access publishing facilitated by Flinders University, as part of the Wiley ‐ Flinders University agreement via the Council of Australasian University Librarians.

## Conflicts of Interest

The authors declare no conflicts of interest.

## Supporting Information

Additional supporting information can be found online in the Supporting Information section.

## Supporting information


**Supporting Information** Table S1: The PRISMA checklist, detailing adherence to established reporting guidelines for systematic reviews and meta‐analyses. Figure S1: The risk‐of‐bias assessment using SYRCLE’s tool for animal studies, including both domain‐level evaluations and overall judgments categorized as low, unclear, or high risk. Table S2: A leave‐one‐out meta‐analysis for the incidence of long‐bone fractures. Table S3: A leave‐one‐out meta‐analysis for the incidence of mice with at least one long‐bone fracture. Table S4: A leave‐one‐out meta‐analysis for maximum load. Table S5: A leave‐one‐out meta‐analysis for trabecular bone volume fraction (BV/TV). Table S6: A leave‐one‐out meta‐analysis for cortical thickness. Table S7: A leave‐one‐out meta‐analysis for stiffness.

## Data Availability

The data that support the findings of this study are available in the Supporting Information of this article.
