# Supplementary material for: Efficacy of Fetal MSC Therapy for Osteogenesis Imperfecta: A Systematic Review and Meta‐Analysis in Mice
Source: Stem Cells Int. 2026 Sep 25;2026:7815746. doi: 10.1155/sci/7815746 (PMC13614066; doi:10.1155/sci/7815746)
Supplement: Supplementary file 1 [file SCI-2026-7815746-s001.docx]

**Table S1.** Preferred Reporting Items for Systematic reviews and Meta-Analyses (PRISMA) checklist.

| **Section/topic** | **#** | **Checklist item** | **Reported on page #** |
| --- | --- | --- | --- |
| **TITLE** | | |  |
| Title | 1 | Identify the report as a systematic review, meta-analysis, or both. | 1 |
| **ABSTRACT** | | |  |
| Structured summary | 2 | Provide a structured summary including, as applicable: background; objectives; data sources; study eligibility criteria, participants, and interventions; study appraisal and synthesis methods; results; limitations; conclusions and implications of key findings; systematic review registration number. | 2 |
| **INTRODUCTION** | | |  |
| Rationale | 3 | Describe the rationale for the review in the context of what is already known. | 3 |
| Objectives | 4 | Provide an explicit statement of questions being addressed regarding participants, interventions, comparisons, outcomes, and study design (PICOS). | 3 |
| **METHODS** | | |  |
| Protocol and registration | 5 | Indicate if a review protocol exists, if and where it can be accessed (e.g., Web address), and, if available, provide registration information, including registration number. | N/A |
| Eligibility criteria | 6 | Specify study characteristics (e.g., PICOS, length of follow-up) and report characteristics (e.g., years considered, language, publication status) used as criteria for eligibility, giving rationale. | 5 |
| Information sources | 7 | Describe all information sources (e.g., databases with coverage dates, contact with study authors to identify additional studies) in the search and the date last searched. | 4 |
| Search | 8 | Present a complete electronic search strategy for at least one database, including any limits used, so it could be repeated. | 6 |
| Study selection | 9 | State the process for selecting studies (i.e., screening, eligibility, included in systematic review, and, if applicable, included in the meta-analysis). | 6 |
| Data collection process | 10 | Describe data extraction method from reports (e.g., piloted forms, independently, in duplicate) and any processes for obtaining and confirming data from investigators. | 6 |
| Data items | 11 | List and define all variables for which data were sought (e.g., PICOS, funding sources) and any assumptions and simplifications made. | 6 |
| Risk of bias in individual studies | 12 | Describe methods used for assessing risk of bias of individual studies (including specification of whether this was done at the study or outcome level), and how this information will be used in any data synthesis. | 5 |
| Summary measures | 13 | State the principal summary measures (e.g., risk ratio, difference in means). | 6 |
| Synthesis of results | 14 | Describe the methods of handling data and combining results of studies, if done, including measures of consistency (e.g., I^2^) for each meta-analysis. | 6 |

*From:*  Moher D, Liberati A, Tetzlaff J, Altman DG, The PRISMA Group (2009). Preferred Reporting Items for Systematic Reviews and Meta-Analyses: The PRISMA Statement. PLoS Med 6(7): e1000097. doi:10.1371/journal.pmed1000097

**Figure S1**. Risk of Bias assessment using SYRCLE's tool for animal studies. Domain-level and overall risk-of-bias judgments for each cohort study. Judgments are categorized as low, unclear, and high, corresponding to green, yellow, and red, respectively.

**Table S2**. Leave-one-out (LOO) meta-analysis for incidence of long-bone fractures.

|  | **RR** | **95%-CI** | **p-value** | **I^2^ (%)** |
| --- | --- | --- | --- | --- |
| **Omitting Jones et al. (2014)** | 0.29 | [0.00; 23.83] | 0.1739 | 45.5 |
| **Omitting Guillot et al. (2008)** | 0.27 | [0.01; 5.47] | 0.1137 | 4.0 |
| **Omitting Ranzoni et al (2016)** | 0.37 | [0.09; 1.49] | 0.0700 | 0 |
| **Original random effects model** | 0.30 | [0.13; 0.72] | 0.0273 | 1.6 |

**Table S3**. Leave-one-out meta-analysis for the incidence of mice with at least one long-bone fracture.

|  | **RR** | **95%-CI** | **p-value** | **I^2^ (%)** |
| --- | --- | --- | --- | --- |
| **Omitting Jones et al. (2014)** | 0.51 | [0.00; 79.34] | 0.3385 | 84.1 |
| **Omitting Guillot et al. (2008)** | 0.40 | [0.05; 3.21] | 0.1126 | 0 |
| **Omitting Ranzoni et al (2016)** | 0.60 | [0.03; 11.44] | 0.2725 | 60.0 |
| **Original random effects model** | 0.50 | [0.18; 1.37] | 0.0976 | 71.2 |

**Table S4.** Leave-one-out meta-analysis for maximum load (N).

|  | **SMD** | **95%-CI** | **p-value** | **I^2^ (%)** |
| --- | --- | --- | --- | --- |
| **Omitting Jones et al. (2014)** | 1.03 | [-12.70; 14.75] | 0.5160 | 91.9 |
| **Omitting Ranzoni et al (2016)** | -0.14 | [-0.99; 0.72] | 0.2930 | 0 |
| **Omitting Vanleene et al (2011)** | 0.94 | [-13.70; 15.57] | 0.5647 | 94.4 |
| **Original random effects model** | 0.60 | [-2.57; 3.77] | 0.5010 | 89.4 |

**Table S5.** Leave-one-out meta-analysis for trabecular bone density fraction (BV/TV) (%).

|  | **SMD** | **95%-CI** | **p-value** | **I^2^ (%)** |
| --- | --- | --- | --- | --- |
| **Omitting Jones et al. (2014)** | -0.10 | [-8.61; 8.41] | 0.9068 | 75.0 |
| **Omitting Ranzoni et al (2016)** | 0.35 | [-14.05; 14.74] | 0.8116 | 87.7 |
| **Omitting Vanleene et al (2011)** | 0.99 | [-4.76; 6.74] | 0.2733 | 9.9 |
| **Original random effects model** | 0.40 | [-2.45; 3.24] | 0.6102 | 79.2 |

**Table S6**. Leave-one-out meta-analysis for cortical thickness (mm).

|  | **SMD** | **95%-CI** | **p-value** | **I^2^ (%)** |
| --- | --- | --- | --- | --- |
| **Omitting Jones et al. (2014)** | 1.62 | [-30.19; 33.43] | 0.6342 | 88.5 |
| **Omitting Guillot et al. (2008)** | -0.62 | [-0.80; -0.44] | 0.0146 | 0 |
| **Omitting Vanleene et al (2011)** | 1.63 | [-29.98; 33.24] | 0.6309 | 88.6 |
| **Original random effects model** | 0.65 | [-5.99; 7.28] | 0.7159 | 77.7 |

**Table S7**. Leave-one-out meta-analysis for stiffness (N/mm).

|  | **SMD** | **95%-CI** | **p-value** | **I^2^ (%)** |
| --- | --- | --- | --- | --- |
| **Omitting Jones et al. (2014)** | 1.16 | [-18.87; 21.19] | 0.5955 | 95.6 |
| **Omitting Ranzoni et al (2016)** | -0.51 | [-1.48; 0.47] | 0.0957 | 0 |
| **Omitting Vanleene et al (2011)** | 1.07 | [-20.00; 22.13] | 0.6354 | 96.7 |
| **Original random effects model** | 0.57 | [-4.04; 5.17] | 0.6494 | 93.8 |

Abbreviations: RR – Risk Ratio; SMD – Standardised mean difference; 95%-CI – 95% confidence interval.
